# Fragment-based screening identifies molecules targeting the substrate-binding ankyrin repeat domains of tankyrase

**DOI:** 10.1038/s41598-019-55240-5

**Published:** 2019-12-13

**Authors:** Katie Pollock, Manjuan Liu, Mariola Zaleska, Mirco Meniconi, Mark Pfuhl, Ian Collins, Sebastian Guettler

**Affiliations:** 1 Divisions of Structural Biology & Cancer Biology, The Institute of Cancer Research (ICR), London, SW7 3RP United Kingdom; 2 Division of Cancer Therapeutics, The Institute of Cancer Research (ICR), London, SW7 3RP United Kingdom; 3 School of Cardiovascular Medicine and Sciences and Randall Centre, King’s College London, Guy’s Campus, London, SE1 1UL United Kingdom; 4 Present Address: Cancer Research UK Beatson Institute, Drug Discovery Programme, Glasgow, G61 1BD United Kingdom

**Keywords:** Solution-state NMR, High-throughput screening

## Abstract

The PARP enzyme and scaffolding protein tankyrase (TNKS, TNKS2) uses its ankyrin repeat clusters (ARCs) to bind a wide range of proteins and thereby controls diverse cellular functions. A number of these are implicated in cancer-relevant processes, including Wnt/β-catenin signalling, Hippo signalling and telomere maintenance. The ARCs recognise a conserved tankyrase-binding peptide motif (TBM). All currently available tankyrase inhibitors target the catalytic domain and inhibit tankyrase’s poly(ADP-ribosyl)ation function. However, there is emerging evidence that catalysis-independent “scaffolding” mechanisms contribute to tankyrase function. Here we report a fragment-based screening programme against tankyrase ARC domains, using a combination of biophysical assays, including differential scanning fluorimetry (DSF) and nuclear magnetic resonance (NMR) spectroscopy. We identify fragment molecules that will serve as starting points for the development of tankyrase substrate binding antagonists. Such compounds will enable probing the scaffolding functions of tankyrase, and may, in the future, provide potential alternative therapeutic approaches to inhibiting tankyrase activity in cancer and other conditions.

## Introduction

**Figure 1:**
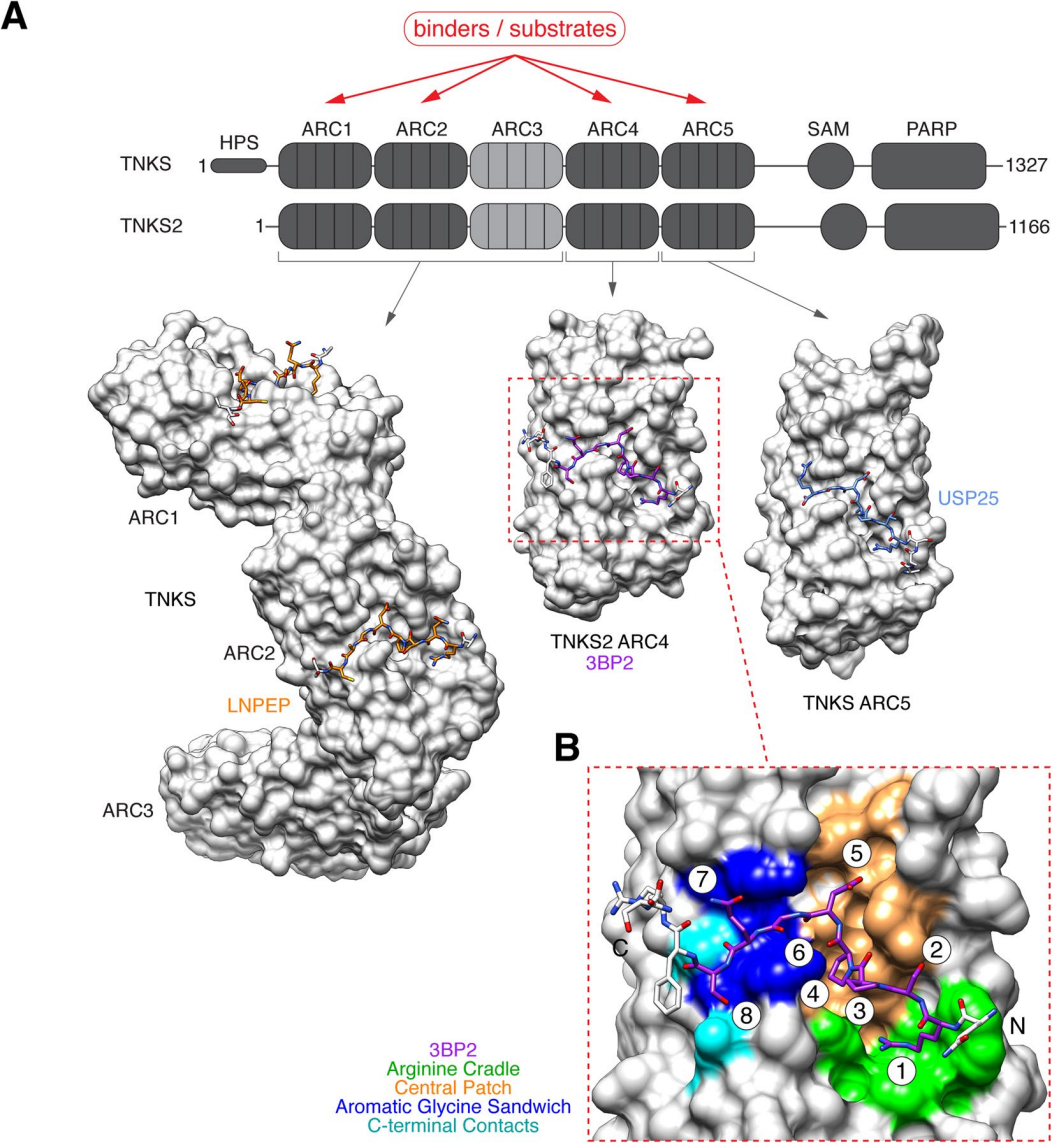
**(A)** Domain organisation of human tankyrase enzymes. Two tankyrase paralogues (TNKS, TNKS2) share an overall sequence identity of 82% (83% across ARCs, 74% across SAM domains, 94% across PARP domains). The ARCs comprise the substrate/protein recognition domain. Several examples of crystal structures of human tankyrase ARCs bound to tankyrase-binding motif (TBM) peptides are shown: TNKS ARC1-3 bound to TBM peptide from LNPEP^48^ (PDB code 5JHQ), TNKS2 ARC4 bound to TBM peptide from 3BP2^4^ (3TWR), TNKS ARC5 bound to TBM peptide from USP25^81^ (5GP7). **(B)** Details of the interaction of TNKS2 ARC4 with a 3BP2 TBM peptide^4^ (3TWR, modified from reference 4). Four TBM peptide-binding hotspots are shown: the “arginine cradle” (green), “central patch” (orange), “aromatic glycine sandwich” (blue), and “C-terminal contacts” (cyan). TBM octapeptide amino acid positions are numbered.

Tankyrase enzymes (TNKS/ARTD5 and TNKS2/ARTD6; simply referred to as ‘tankyrase’ from here on; Fig. 1A) are poly(ADP-ribose)polymerases (PARPs) in the Diphtheria-toxin-like ADP-ribosyltransferase (ARTD) family^1,2^. PARPs catalyse the processive addition of poly(ADP-ribose) (PAR) onto substrate proteins, which can either directly regulate acceptor protein function or serve as docking platform for PAR-binding proteins that mediate downstream signalling events^3^. Given the diversity of tankyrase binders and substrates, tankyrase impinges on a wide range of cellular functions^2,4–6^. These include Wnt/β-catenin signalling^7–10^, telomerase-dependent telomere lengthening^11,12^, sister telomere resolution during mitosis^13,14^, the control of glucose homeostasis^15–17^, mitotic spindle assembly^18,19^, DNA repair^20,21^, and the regulation of the tumour-suppressive Hippo signalling pathway^22–24^. Silencing of tankyrase elicits synthetic lethality in BRCA1/2-deficient cancer cells^25^. Given these links of tankyrase to disease-relevant processes, tankyrase has gained attention as a potential therapeutic target^2,26^.

Many mechanistic aspects of tankyrase function have been revealed by studying its role in the Wnt/β-catenin pathway^10^. Tankyrase promotes Wnt/β-catenin signalling by PARylating AXIN (axis inhibition protein 1/2)^7^, a central component of the multi-protein β-catenin destruction complex, which initiates the degradation of the transcriptional co-activator β-catenin under low-Wnt conditions^27^. PARylation either induces the PAR-dependent ubiquitination and degradation of AXIN^7,28–30^, or promotes the Wnt-induced transformation of the destruction complex into a signalosome complex incapable of initiating β-catenin degradation^8,31^. Tankyrase thus sensitises cells to incoming Wnt signals^32,33^. The Wnt/β-catenin pathway is dysregulated in approximately 90% of colorectal cancer cases^34^. Inhibiting tankyrase has been explored as a strategy to re-tune oncogenically dysregulated Wnt/β-catenin signalling in cancers with mutations in the tumour suppressor and destruction complex component APC (adenomatous polyposis coli)^10,35–40^. Whilst tankyrase catalytic inhibitors can suppress tumour cell growth, *in-vivo* studies have also pointed to different degrees of tankyrase-inhibitor-induced intestinal toxicity in mice^35,39,41^. The precise molecular mechanisms by which tankyrase controls Wnt/β-catenin signalling, how tankyrase inhibition can restore oncogenically dysregulated signalling, and the basis of tankyrase inhibitor toxicity are incompletely charted. This warrants the development of different chemical probes to modulate tankyrase function.

To date, drug discovery efforts on tankyrase have focused on inhibiting the catalytic PARP domain^2,10,42,43^. Catalytic inhibition of tankyrase has complex consequences. As well as inhibiting substrate PARylation, catalytic inhibitors prevent tankyrase auto-PARylation and therefore subsequent PAR-dependent ubiquitination and degradation of tankyrase itself^7^. Consequently, tankyrase catalytic inhibition typically leads not only to the accumulation of many of its substrates but also of tankyrase^6,7,35,37,40^. The accumulation of tankyrase and its substrates may further accentuate catalysis-independent functions of tankyrase, which have been emerging recently. One such example is our surprising observation that tankyrase can promote Wnt/β-catenin signalling independently of its catalytic PARP activity, at least when tankyrase levels are high^9^. Under these conditions, tankyrase catalytic inhibitors do not completely block tankyrase-driven β-catenin-dependent transcription, pointing to both catalytic and non-catalytic (scaffolding) functions of tankyrase. Tankyrase scaffolding functions depend on tankyrase’s substrate-binding ankyrin repeat clusters (ARCs) and the polymerisation function of its sterile alpha motif (SAM) domain (see Fig. 1A)^9^. Tankyrase auto-PARylation has been proposed to limit tankyrase polymerisation^44^. Tankyrase catalytic inhibition may therefore induce its hyperpolymerisation, which may further promote scaffolding functions. Scaffolding functions of tankyrase likely extend beyond Wnt/β-catenin signalling: not all tankyrase binders are also PARylated, and non-catalytic roles of tankyrase in other processes have been proposed^4,5,45,46^.

Unravelling the complexity of tankyrase’s catalytic vs. non-catalytic functions will require novel tool compounds that block tankyrase-dependent scaffolding. We therefore set out to identify and characterise small molecule fragments that bind to the tankyrase ARC domains, as a first step towards the discovery of compounds capable of blocking the interaction of tankyrase binders and substrates with the ARC domains of tankyrase.

Tankyrase contains five N-terminal ankyrin repeat clusters (ARCs), four of which, namely ARCs 1, 2, 4 and 5, can recruit binders and substrates^4,47,48^ (Fig. 1A). ARCs bind conserved but degenerate six- to eight-amino-acid peptide motifs, termed the tankyrase-binding motif (TBM, consensus R-X-X-[small hydrophobic or G]-[D/E/I/P]-G-[no P]-[D/E])^4,49^. Insertions between the residues typically found at positions 1 and 4 can give rise to “non-canonical” TBMs that span more than six or eight amino acids^50,51^. Depending on the binding partner, ARCs can be functionally redundant, at least at the level of substrate recruitment^4^, or collaborate in a combinatorial fashion, engaging preferred sets of ARCs in recruiting multivalent tankyrase binders such as AXIN^48^. The TBM-binding pocket contains several binding hotspots (Fig. 1B). An “arginine cradle” forms the binding site for the TBM’s essential arginine residue at position 1. The “central patch” accomodates diverse interactions, including hydrophobic contacts with a small hydrophobic residue at TBM position 4 and contact sites for the residue at TBM position 5. An “aromatic glycine sandwich” refers to the invariant glycine at TBM position 6 sandwiched between two aromatic residues^4^.

Mutation of the TBM binding sites in the ARCs abrogates tankyrase’s ability to bind substrates and drive Wnt/β-catenin signalling^9,48^. As a further proof of concept for the feasibility of targeting tankyrase via the ARCs, a sequence-optimised^4^, cell-permeating stapled TBM peptide can compete with AXIN for tankyrase binding and suppress the Wnt-induced expression of a β-catenin-responsive reporter gene in HEK293 cells^52^. Given the uniqueness of ARCs within the PARP family and the high degree of conservation across both TNKS and TNKS2, interfering with substrate binding would likely provide high target specificity and inhibition of both TNKS and TNKS2, many of whose functions are redundant^6,53^.

Herein, we report the identification and characterisation of fragments that bind to tankyrase ARCs at the same site as the TBM peptides. The identified fragments provide a starting point for the development of potent, cell-active tankyrase substrate binding antagonists.

## Results

### Essentiality of the TBM arginine residue

We first considered a peptidomimetic approach to develop TBM peptides into more potent, stable and drug-like competitors of the ARC:TBM interaction. Given the anticipated impairment of cell permeability by the N-terminal TBM arginine, we investigated whether the guanidine group could be substituted. To prioritise synthesis efforts, we followed an *in-silico* docking approach^54^, exploring the importance of the positive charge and hydrogen bonding interactions, linker lengths/flexibility and side chain geometry (see Supplementary Materials and Methods for details). From commercially available side chain alternatives, we identified five potential candidates for R replacements: 1H-imidazole-5-pentanoic acid, 1H-imidazole-1-pentanoic acid, 7-aminoheptanoic acid, D-arginine and L-citrulline (Supplementary Fig. 1). We next synthesised 3BP2 TBM octapeptides, incorporating the five arginine substituents at position 1, followed by fluorescence polarisation (FP) assays to assess competition of the peptides with a Cy5-labelled TBM peptide probe (Supplementary Fig. 1). We used a 16-mer TBM peptide (LPHLQ**RSPPDGQS**FRSW, W introduced to measure A_280_) derived from the model substrate 3BP2, a signalling adapter protein^4^, as a positive control for a competitor, and a corresponding non-binding TBM peptide bearing a glycine-to-arginine substitution at position 6^4^ as a negative control. Whilst we observed no binding for the G6R negative control, we measured an IC_50_ of 22 μM for the 3BP2 16-mer peptide (Supplementary Fig. 1). An 8-mer **RSPPDGQS** TBM peptide displayed an IC_50_ of 34 μM. Substituting L-arginine for D-arginine caused a five-fold drop in potency to 175 μM, highlighting the importance of side chain geometry. Both imidazole moiety peptides displayed IC_50_ values in the 500 μM range. The 7-aminoheptanoic acid and citrulline peptides showed poor competition and precipitation at high concentrations (Supplementary Fig. 1). In conclusion, these observations demonstrated that the essential arginine residue of the TBM cannot be easily substituted.

### Primary fragment screens

Given the anticipated challenges associated with replacing the TBM arginine residue, we pursued a fragment screening strategy to sample a wide range of chemical space, with the aim of finding novel, ligand-efficient small molecules that target tankyrase ARCs and to identify alternative binding ‘hotspots’ away from the “arginine cradle” of the TBM binding site. We screened The Institute of Cancer Research (ICR) fragment library^55^ in parallel against TNKS2 ARC5 using differential scanning fluorimetry (DSF), and TNKS2 ARC4 using ligand-observed nuclear magnetic resonance (NMR) spectroscopy techniques. We used the 16-mer 3BP2 TBM peptide and its non-binding mutant variant as positive and negative controls, respectively.

### Primary fragment screening by DSF

**Figure 2:**
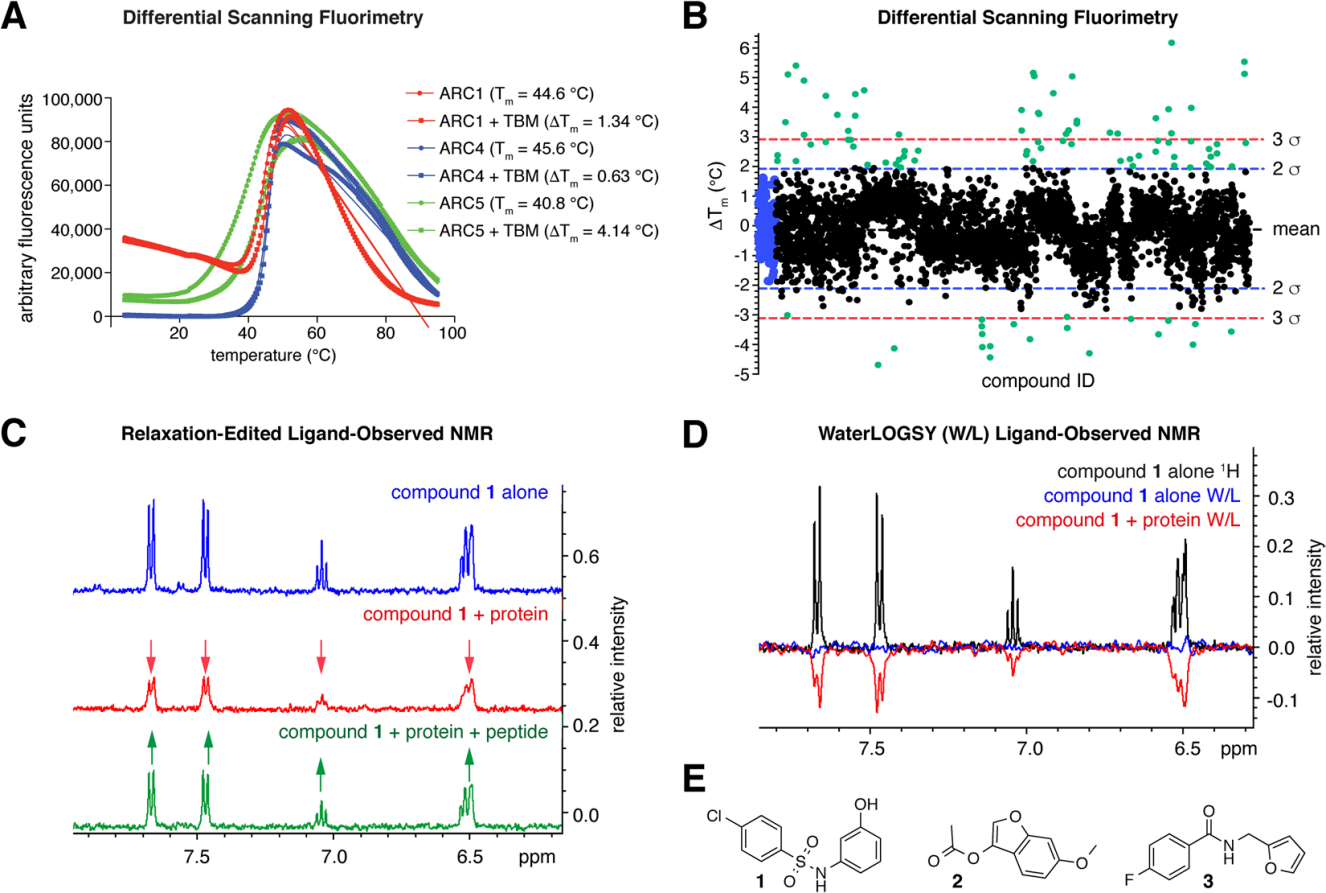
**(A)** Differential scanning fluorimetry (DSF, a.k.a. ThermoFluor) assessment of TNKS2 ARCs 1, 4 and 5 shows that TNKS2 ARC5 is the least stable among these ARCs and experiences the highest degree of thermal stabilisation upon 3BP2 TBM peptide binding. **(B)** Fragment screen against TNKS2 ARC5 by DSF: the graph shows ΔT_m_ (from the IP method) plotted vs. compound ID for both replicates. DMSO-only controls are coloured blue; hit fragments are coloured green. Lines correspond to the mean, and 2 or 3 standard deviations outside the mean. **(C)** Example of relaxation-edited spectra for hit compound **1**. Signals are reduced in the presence of protein (red), indicating ARC binding, and recovered upon TBM peptide addition (green), indicating competition. **(D)** Example of waterLOGSY spectra for hit compound **1**, showing a negative NOE signal when protein is added (red). Buffer (HEPES) signals were phased as positive peaks in our waterLOGSY spectra. **(E)** Structures of hit compounds from the DSF screen that bound both TNKS2 ARCs 4 and 5 and were competitive with a TBM peptide, as measured by relaxation-edited ligand-observed NMR.

Our pilot studies showed that among all TNKS2 single ARCs that we could produce recombinantly (ARCs 1, 4 and 5)^49^, ARC5 displayed the lowest melting temperature (T_m_) and the largest shift in melting temperature upon addition of the 3BP2 TBM peptide (ΔT_m_) (Fig. 2A). Therefore, we chose TNKS2 ARC5 for screening by DSF, anticipating the largest signal window for measuring changes in T_m_ upon fragment binding. DMSO concentrations up to 10% of total sample volume had a negligible effect on TNKS2 ARC5 T_m_ (Supplementary Fig. 2A). We explored the stabilisation of TNKS2 ARC5 by the abovementioned TBM peptide derivatives and found a good correlation between the DSF data and the FP data obtained with TNKS2 ARC4, further demonstrating the suitability of the DSF assay (Supplementary Fig. 2B).

We screened 1869 compounds in duplicate at a concentration of 500 μM, which we considered a reasonable compromise between having a sufficiently high concentration to identify weak binders while minimising the likelihood of false positives through fragment precipitation/aggregation and non-specific binding. The final DMSO concentration was 5%. We calculated melting temperatures using both the inflection point and the maximum peak of first derivative data methods. Both methods generally agreed, unless the melt curve was biphasic or misshapen, with slightly lower variability for the first derivative T_m_ determination method (see Materials and Methods for experimental and analysis details).

We determined the melting temperature for the unbound ARC (T_m, 0_) from the mean of 12 reference melting curves per plate, with 5% DMSO only. We calculated the change in melting temperature (ΔT_m_) by subtracting the mean T_m, 0_ from T_m, compound_. We tested compounds in duplicate, defining fragments as hits if they conferred a ΔT_m_ outside two standard deviations (2σ) from the mean, in one or both replicates. To check for consistency between plates, we ran triplicate peptide controls; however, we excluded peptide ΔT_m_ values from the calculation of the mean ΔT_m_ to avoid skewing the results. We observed mean ΔT_m_ values (IP/1^st^ derivative methods) of −0.127/−0.331 °C and σ as 0.997/1.19 °C. A hit cut-off of 2σ gave absolute shifts of +1.87/+2.05 °C for compounds that stabilised and −2.13/−2.71 °C for compounds that destabilised the ARC (Fig. 2B).

We assessed the robustness of the assay for screening. The standard deviation for both the DMSO-only (T_m, 0_) and 3BP2 peptide positive control (T_m, peptide_) melting temperatures across all plates was approximately 1 °C, indicating that any shifts below 1 °C may be attributable to noise. We calculated the Z factor (Z’) using the mean melting temperature and σ for the whole fragment library, with the DMSO-only samples as the baseline and 3BP2 TBM peptide samples as positive controls. A value of Z’ = 0.9 was obtained, indicating that the assay was robust.

We prioritised hits that stabilised TNKS2 ARC5 if they had a change in melting temperature of greater than 1.8 °C in at least one replicate (both ΔT_m_(IP) and ΔT_m_(1st derivative) methods of analysis), and de-prioritised those that showed a substantial discrepancy between ΔT_m_(IP) and ΔT_m_(1st derivative) values, indicating an unusual melting curve shape. Negative shift hits that destabilised the protein were only taken forward if they were significant in both replicates, and with both methods of ΔT_m_ analysis. The higher stringency was applied as molecules that destabilise the protein can be harder to advance and develop into lead-like compounds^56,57^. We thus progressed 56 hits into validation assays. Of these, 48 conferred a positive thermal shift and stabilised TNKS2 ARC5; 8 had a negative thermal shift and destabilised the protein. We next assessed compound purity and structural integrity of hits from the DSF screen by liquid-chromatography-mass-spectrometry (LC-MS) and measured their solubility by NMR. Five compounds failed the LC-MS quality control, and eight were of insufficient solubility by NMR (<100 μM in aqueous buffer with 5% DMSO). Two compounds didn’t contain any aromatic protons (required for our NMR solubility assay), and an additional two were no longer commercially available for re-purchase. A total of 17 of compounds were therefore excluded from further analysis. An *in-silico* pan assay interference compounds (PAINS) screen was applied to the hit fragments to highlight any possible issues in carrying the hits forward^58^. No compounds were flagged as problematic in the PAINS screen.

### Fragment binding validation for DSF hits

39 hits from the DSF screen were suitable for follow-up by ligand-observed NMR methods. We re-purchased fragments and performed T2 relaxation-edited (CPMG-edited) and waterLOGSY experiments for each fragment with TNKS2 ARC5. We explored saturation transfer difference (STD) NMR, also using TNKS2 ARC5, but the assay was not sensitive enough to produce a reliable binding signal, likely due to the relatively small size of a single ARC protein (data not shown). High ligand concentrations were required to achieve sufficient signal, which in turn could lead to false-positive hits due to non-specific binding.

Using the relaxation-edited assay, we tested each fragment in three independent measurements (Fig. 2C), unless we obtained two negative results (non-binding) in the first two experiments. We next used waterLOGSY to further evaluate compounds that showed a substantial decrease (>15% reduction in peak integrals) upon protein addition in at least one out of three relaxation-edited experiments. We classified fragments with a negative NOE signal in waterLOGSY as binders (Fig. 2D). As a negative NOE for the compound-only sample could indicate aggregation, we flagged these compounds as potentially problematic. We identified 14 fragments that bound to TNKS2 ARC5 by both relaxation-edited and waterLOGSY methods (0.78% hit rate). We next tested whether binding of these fragments occurred competitively with the 3BP2 TBM peptide, and also if they bound to TNKS2 ARC4, as competition with peptide binders at various different ARCs will be a prerequisite for an efficient substrate binding antagonist. Three fragments (**1**, **2**, **3**) bound to both TNKS2 ARC4 and ARC5 and were competitive with the TBM peptide (0.16% hit rate) (Fig. 2E). Three further fragments also bound to both ARCs, but were not TBM competitive by NMR.

### Primary fragment screening by NMR

**Figure 3:**
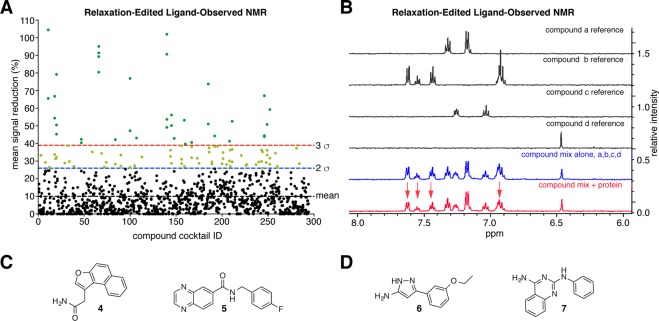
**(A)** Summary of ligand-observed NMR screen, showing percentage of signal change vs. compound cocktail ID. Lines correspond to the mean, and 2 or 3 standard deviations outside the mean. **(B)** Example data for a cocktail of 4 compounds, containing one hit (compound b) and three non-binding fragments. **(C)** Structures of hit fragments uniquely identified in the NMR screen and TBM-competitive, as assessed by relaxation-edited NMR. **(D)** Structures of compounds that were identified as hits in both the DSF and NMR primary screens.

The hit rate for compounds confirmed to bind TNKS2 ARC5 as evaluated by NMR was relatively low, at 0.78%, and only 0.16% for fragments binding TNKS2 ARC4 and 5 competitively with a TBM peptide. Different screening assays often identify distinct hit fragments^59^. There is no consensus on the most appropriate assays to use for fragment screening, especially against challenging targets such as protein-protein interactions. Often several orthogonal methods are used in series to narrow down fragment hits, or a combination of biophysical and biochemical assays to exclude false positives and identify binders that modulate protein activity^55^. We therefore carried out an additional primary screen using T2 relaxation-edited ligand-observed NMR on TNKS2 ARC4, probing a subset of molecules from the ICR fragment library that was compatible with NMR. We screened 1100 compounds in pools of four structurally dissimilar molecules with non-overlapping proton resonances (Fig. 3A,B)^60^. We split the top 100 hits into two groups for individual re-testing: those with a signal change >39% (3σ, 35 compounds), and those with a signal change of 26–39% reduction (2–3σ, 65 compounds) (Fig. 3A). We tested fragments of the first hit group (>3σ) individually using the T2 relaxation-edited NMR assay. We tested those of the second hit group (2–3σ) in a waterLOGSY NMR experiment, reasoning that this may rescue any genuine binders with a relatively small signal in the relaxation-edited assay. Nine out of 35 compounds from hit set 1 displayed a significant intensity change (≥26% reduction) upon protein addition when tested individually. We confirmed seven out of 65 compounds from hit set 2 to bind in the waterLOGSY assay. We tested these 16 compounds in further T2 relaxation-edited and waterLOGSY experiments, and in competition with the 3BP2 TBM peptide. Of the 16 compounds, two (**4** and **5**) were competitive with the TBM peptide by relaxation-edited NMR; one compound (**5**) also showed peptide competition by waterLOGSY (Fig. 3C).

In addition to the hits identified uniquely by either the DSF or NMR screens, the two orthogonal screens also shared two common hits (compounds **6** and **7**, Fig. 3D). Retrospective analysis of the DSF screening data revealed that compound **5** was excluded during the DSF analysis due to poorly shaped, biphasic melt curves. This resulted in large discrepancies between ΔT_m_ values calculated by the inflection point (1.6 °C) and 1^st^ derivative methods (8.1 °C) when the second peak was used to calculate T_m_. We also discounted other compounds for poor melt curve shape; however, none of these were identified in the orthogonal NMR-based screen.

### Fragment hit validation and K_d_ determination

We next tested validated hits from both the DSF and NMR screens against TNKS2 ARC4 using protein-observed NMR. This included 16 compounds in total: 14 fragments identified by DSF and confirmed to bind TNKS2 ARC5 by both relaxation-edited and waterLOGSY methods and two unique fragments identified by NMR and confirmed to bind TNKS2 ARC4 by both ligand-observed NMR methods. We used the 3BP2 TBM peptide as a positive control. To directly identify fragment binding sites on the ARC, we performed a full backbone and partial side-chain assignment of TNKS2 ARC4, doubly labelled with ^15^N and ^13^C isotopes. The assignment details have been reported elsewhere^61^. The control peptide induced significant chemical shift perturbations (CSPs), indicative of peptide binding in both a fast and slow kinetic regime (Supplementary Fig. 3). Interestingly, among the residues that constitute the TBM binding site on the ARC, residues that exhibited the slow-exchange binding mode are part of the “central patch” (D521, S527, F532, D556, L560, H564, N565, S568) and the “aromatic glycine sandwich” (G535, Y536, Y569); a single residue from the “arginine cradle” (F593) displayed slow exchange. This suggests that these areas constitute key TBM:ARC interaction hotspots. Residues that exhibited the fast-exchange binding mode were from the “arginine cradle” (D589, W591, E598) and the “C-terminal contacts” (H571, K604) (Supplementary Fig. 3). The different binding regimes may distinguish primary interaction hotspots that are engaged robustly when a peptide is first recruited (slow exchange) from secondary binding sites in the ARC that become occupied once primary binding hotspots are engaged; these may be more dynamic (fast exchange).

We then titrated compounds against ^15^N-labelled TNKS2 ARC4, initially at protein:compound ratios of 1:1 and 1:3. Two fragments (**3** and **5**) induced significant CSPs (data not shown); we used these to perform an eight-point titration and observed concentration-dependent CSPs (Supplementary Fig. 4A,B). We confirmed that the CSPs were not caused by pH changes during the titration by measuring the pH of the peptide and fragment stocks (at 3 mM) in assay buffer. Consistent with TBM-competitive binding of fragments **3** and **5**, we identified several peaks that shifted in both fragment and 3BP2 TBM peptide titrations. Compound **5** caused more significant perturbations than compound **3**, and they all exhibited a fast-exchange regime (Supplementary Fig. 4C). Peaks that moved significantly (CSPs > Δδ_tot_ + 2σ) upon addition of compound **5** are part of the “central patch” and “aromatic glycine sandwich” (S527, F532, G535, Y536, N565; see Supplementary Fig. 4C). However, the solubility of fragments **3** and **5** in assay buffer limited the maximum concentration achievable, and so complete saturation was not reached. This confounded affinity measurements and more extensive analyses of the fragment binding sites by protein-observed NMR.

### Fragment analogue SAR

We next sought close structural analogues of hit fragments **3** and **5** to gain early insights into structure-activity relationships (SAR) and binding modes of hit fragments. We initially tested the analogues using both relaxation-edited and waterLOGSY NMR assays against TNKS2 ARC4 (Table 1), followed by protein-observed NMR if binding was detected in both ligand-observed NMR experiments. Compounds displaying negative NOE signals in the waterLOGSY assay upon protein addition were classed as binders; however, compounds that displayed negative NOE signals in the absence of protein were flagged as potential aggregators. Compounds that displayed no NOE signal were classed as non-binders.

**Table 1:** Analogues of compounds **3** and **5** tested by ligand-observed and protein-observed NMR.

No	Structure	Solubility (μM)	T2 relaxation-edited	waterlogsy	^15^N NMR
compound **5** and analogues					
5		410	33.5% reduction, competitive	negative NOE	substantial CSPs
8		140	25.4% reduction, competitive	negative NOE	substantial CSPs
9		650	42.4% reduction, competitive	one peak has negative NOE	substantial CSPs
10		630	0% reduction	negative NOE even for compound alone	no CSPs
11		n.d.	0% reduction	no NOE	n.d.
12		< 50	Not soluble	n.d.	n.d.
13		410	0% reduction	negative NOE even for compound alone	n.d.
14		635	0% reduction	no NOE	n.d.
15		470	20% reduction	negative NOE even for compound alone	no CSPs
compound **3** and analogues					
3		220	52.6% reduction	negative NOE	substantial CSPs
16		930	25% reduction	no NOE	n.d.
17		n.d.	0% reduction	no NOE	n.d.
18		900	16% reduction	no NOE	n.d.
19		255	0% reduction	no NOE	n.d.
20		n.d.	0% reduction	no NOE	n.d.
21		n.d.	0% reduction	no NOE	n.d.
22		n.d.	0% reduction	no NOE	n.d.

Compounds **17, 18** and **20**–**22** were already present in the fragment library and were therefore not re-tested. The original screening hits (**3** and **5**) are shown as references. “n.d.”, not determined.

Substituting the para-fluorine of compound **5** for a methyl group preserved binding (**8**), as did replacement of the entire Ar-F group with a furan (**9**). Analogue **9** showed increased solubility over original hits **3** and **5**. Contracting the quinoxaline ring by one carbon atom to a benzimidazole (**10**) abrogated binding. Substitution of the quinoxaline moiety by a triazolopyrimidine (**11**) also abrogated binding. Shortening the amide linker by one carbon (**12**) limited solubility. Increasing the linker length by one carbon (**13**) abolished binding in the relaxation-edited NMR assay; however, it also resulted in a strong waterLOGSY signal. We also observed a strong waterLOGSY signal for compound **13** in the absence of protein, indicating that this compound may aggregate. Methylating the amide nitrogen of compound **9** was not tolerated (**14**). Additionally substituting the quinoxaline ring at positions 2 and 3 with methyl groups (**15**) led to a response in relaxation-edited ligand-observed NMR; however, the waterLOGSY data suggested compound aggregation, and no CSPs were observed in protein-observed NMR. In summary, we demonstrated TNKS2 ARC4 binding activity of several quinoxaline analogues of compound **5**, confirming this hit series and showing that the Ar-F group of compound **5** could be readily substituted.

For the benzamide fragment (**3**), moving the fluorine atom from the para to the meta position (**16**) or adding an ortho-fluorine (**17**) abolished binding. Substitution of the furan for a pyridine (**18**) or Ar-F moiety (**19**) was not tolerated. Reversing or rearranging the amide linker (**20, 21**, **22**) whilst simultaneously changing the furan for a piperidine (**20**), adding a methyl group to the furan ring (**21**) or substituting the para-fluorine for a meta-chlorine (**22**) were also not tolerated.

**Figure 4:**
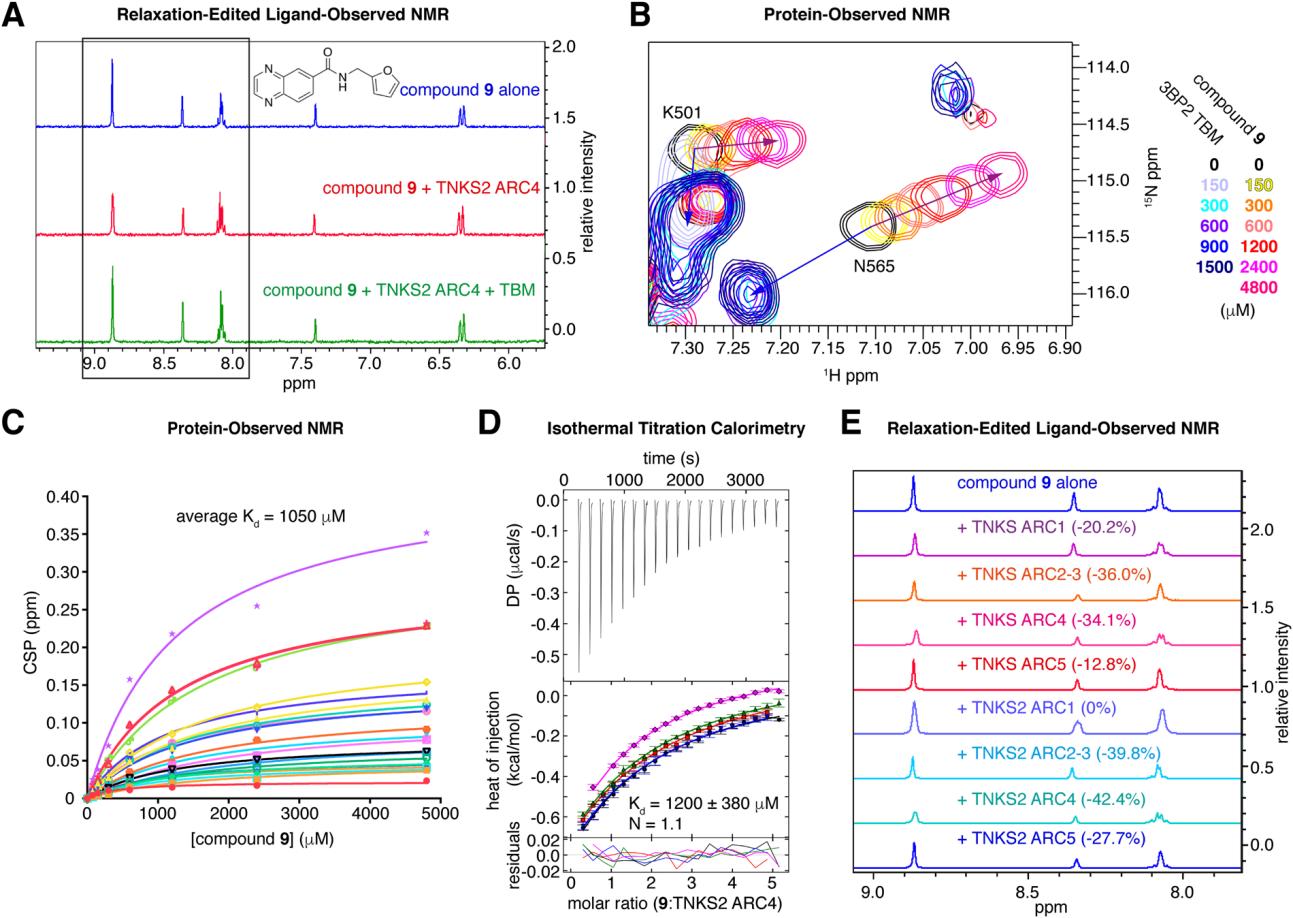
**(A)** Relaxation-edited NMR for compound **9**, showing the largest reduction in peak height for the quinoxaline protons (boxed), indicating that the majority of binding can be attributed to the quinoxaline moiety. **(B)** Protein-observed NMR for TNKS2 ARC4. Example area of superimposed ^1^H-^15^N HSQC NMR spectra, showing the chemical shift perturbations (CSPs) upon TBM peptide or compound **9** titration. **(C)** K_d_ estimate of compound **9** by plotting the CSPs of peaks that moved in a concentration-dependent manner. **(D)** ITC for the titration of compound **9** (5 mM) into TNKS2 ARC4 (200 μM). The K_d_ for compound **9** was calculated to be 1200 ± 380 μM; the stoichiometry of compound **9**:TNKS2 ARC4 was 1.1 (global analysis of n = 5 independent experiments). See Supplementary Fig. 5 for an example titration of compound **9** into buffer. **(E)** Compound **9** binding to TNKS and TNKS2 ARCs was assessed by relaxation-edited NMR. Total reductions in peak area upon ARC addition are indicated.

Compound **9** (Table 1, Fig. 4A) combined features of both fragments **3** and **5**, namely the quinoxaline group of fragment **5**, the amide linker shared by both fragments and the furan group of fragment **3**. We observed that in the relaxation-edited NMR experiments, peaks corresponding to the quinoxaline displayed a larger reduction upon ARC addition than peaks attributed to furan (Fig. 4A). This suggested that the quinoxaline moiety more substantially contributes to the binding, and several analogues of the quinoxaline hit were confirmed to bind to TNKS2 ARC4.

### Fragment binding affinity

The increased solubility of compound **9** compared to compound **5** allowed complete saturation in a protein-observed NMR titration experiment against TNKS2 ARC4, yielding an apparent K_d_ of 1050 μM (Fig. 4B,C). We next used isothermal titration calorimetry (ITC) to confirm the compound **9**:TNKS2 ARC4 binding affinity, titrating the fragment (5 mM, 1% DMSO) into TNKS2 ARC4 (200 μM, 1% DMSO). A global analysis of 5 experiments confirmed the affinity to be in the region of 1 mM (1200 ± 380 μM) with a stoichiometry of 1.1 (Fig. 4D, Supplementary Fig. 5, Table 2).

**Table 2:** Summary of data for fragments confirmed to bind to TNKS2 ARC4 by protein-observed NMR.

no	structure	ΔT_m_ (°C)	NMR solubility (μM)	T2 relaxation-edited (n = 3)	waterLOGSY	^15^N HSQC	NMR K_d_ (μM)	ITC K_d_ (μM)
3		2.5	220	52.6% reduction	binding	CSPs observed	no saturation	—
5		—	410	33.5% reduction	binding	CSPs observed	no saturation	—
9		—	650	42.4% reduction	binding	significant CSPs	1050	1200

### Fragments bind to multiple TNKS and TNKS2 ARCs

The anticipated functional redundancy between ARCs and the existence of two tankyrase paralogues will require efficient substrate binding antagonists to ideally bind all TBM-binding ARCs of both TNKS and TNKS2. The high conservation of the peptide-binding pocket (Supplementary Fig. 6) suggests that this goal should be achievable^4^. We tested binding of compound **9** to all TNKS and TNKS2 ARCs using the relaxation-edited NMR assay (Fig. 4E, Table 3). Compound **9** bound all ARCs, with the exception of TNKS2 ARC1. Given the invariant residue infrastructure of the peptide-binding pocket in ARC1 of TNKS and TNKS2, this observation is difficult to reconcile with available structural information. It is possible that the presence of glycine-sandwiching phenylalanine rather than tyrosine residues in ARC1 of both tankyrases, paired with the presence of a phenylalanine in TNKS2 (F29^TNKS2^) as opposed to a leucine in TNKS (L187^TNKS^) confers this differential behaviour. F29^TNKS2^/L187^TNKS^ sit in an extended hydrophobic pocket “above” the “central patch” and may, directly or indirectly, participate in fragment binding. However, it remains possible that other differences within the N-terminal capping repeat of ARC1, including the β-hairpin linking to the subsequent repeat, are responsible for the observed differential binding. Whilst the low affinity of the current fragments may sensitise them to small differences between the TBM-binding ARCs, further developed molecules will need to be engineered to resist such variability.

**Table 3:** Pan-ARC binding activity of compound **9**, tested by ligand-observed NMR.

ARC construct	T2 relaxation-edited	waterLOGSY
TNKS ARC1 (178–336)	20.2% reduction	negative NOE signal
TNKS ARC2–3 (331–645)	36.0% reduction	negative NOE signal
TNKS ARC4 (646–807)	34.1% reduction	negative NOE signal
TNKS ARC5 (799–958)	12.8% reduction	negative NOE signal
TNKS2 ARC1 (20–178)	0%	no NOE
TNKS2 ARC2–3 (173–487)	39.8% reduction	negative NOE signal
TNKS2 ARC4 (488–649)	42.4% reduction	negative NOE signal
TNKS2 ARC5 (641–800)	27.7% reduction	negative NOE signal

### *In-silico* prediction of potential fragment binding hotspots

**Figure 5:**
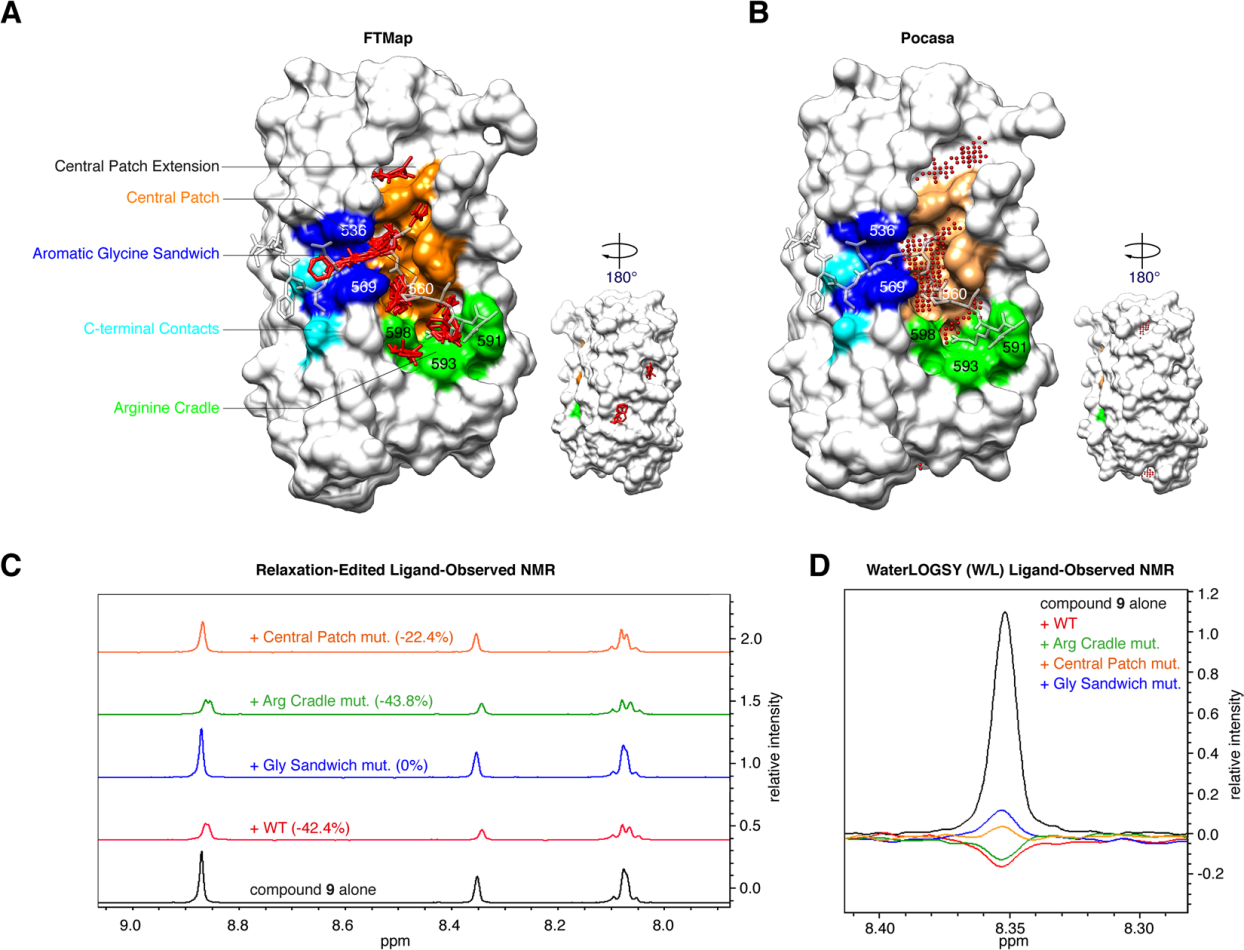
**(A)** Seven fragment binding hotspots on TNKS2 ARC4 predicted by FTMap are in the TBM peptide binding site on the ARC, and one in close vicinity. FTMap analysis was done on TNKS2 ARC4 from the ARC4:3BP2 co-crystal structure^4^ (3TWR). Key residues of the peptide binding site are colour-coded as in Fig. 1B. The TBM peptide from 3BP2 is overlaid in transparent stick representation. The minimum energy hotspot found is in the “central patch” adjacent to the “glycine sandwich”. **(B)** Pocket identification on TNKS2 ARC4 (from the ARC4:3BP2 co-crystal structure, 3TWR) using the Roll algorithm implemented in Pocasa^64^. The three top-ranking pockets are part of the “central patch”, a “central patch extension” and the “arginine cradle”. **(C)** Relaxation-edited NMR of compound **9** with TNKS2 ARC4 peptide binding site mutant variants^4^. Mutation of the “aromatic glycine sandwich” or the “central patch” abolishes or reduces binding of the compound, respectively, whilst binding is unaffected by mutation of the “arginine cradle”. Mutated residues, numbered in (**A**) and (**B**), were as follows: “arginine cradle”, WFE591/593/598AAA; “central patch”, L560W; “aromatic glycine sandwich”, YY536/569AA. **(D)** WaterLOGSY NMR confirms that “glycine sandwich” and “central patch” mutations impair binding of compound **9**.

We next sought to determine the fragment binding site on the ARC. To gain insights into plausible fragment binding sites and identify potential hotspots, we undertook an *in-silico* fragment binding experiment by computational solvent mapping using the FTMap programme^62,63^. FTMap identifies pockets where several different small organic molecule probes bind and cluster together; these consensus sites represent potential hotspots for fragment binding. We docked a set of 16 probe molecules into the crystal structure of TNKS2 ARC4, from the ARC4:3BP2 TBM co-crystal structure^4^. FTMap identified ten areas of probe clustering, seven of which overlapped with the known peptide-binding groove (Fig. 5A). The lowest-energy consensus site, and hence most ligandable pocket identified, was the primarily hydrophobic “central patch” adjacent to the “glycine sandwich”. The second most ligandable site predicted was the “arginine cradle”. These predicted hotspots coincide with the experimentally determined hotspots for TBM peptide binding, based on structural data, site-directed mutagenesis and an amino acid scan of the 3BP2 TBM^4,50^. Another hotspot was detected in an extension to the “central patch”, suggesting that it may be possible to grow fragments in a way that utilises this extended pocket. Of note, this “central patch extension” is occupied by a glycerol molecule in the TNKS2 apo-ARC4 crystal structure^4^. The potential fragment binding sites located by FTMap largely coincide with pockets identified using the program Pocasa, which performs a geometric search based on a three-dimensional grid and rolling probe sphere^64^ (Fig. 5B). The “central patch” and “central patch extension” were the highest-ranked pockets, followed by the “arginine cradle”, with volumes/volume depth values of 126/289, 46/108 and 26/73, respectively.

### Ligand-observed NMR with mutant TNKS2 ARC4 proteins

We used three previously designed TNKS2 ARC4 mutant variants^4^ to explore potential binding determinants for fragment **9**. A WFE591/593/598AAA triple-mutation abrogates three key residues in the “arginine cradle”; YY536/569AA truncates two tyrosine residues that form the “glycine sandwich”, and L560W introduces a bulky residue into the “central patch” that sterically clashes with the TBM peptide (see Fig. 5A,B for locations of the mutated residues). We tested binding of compound **9** to the wild-type and mutant ARCs by ligand-observed NMR, using the relaxation-edited assay (Fig. 5C, Table 4). Whilst mutation of the “arginine cradle” had no effect on fragment binding, mutation of the aromatic residues sandwiching the TBM glycine (YY536/569AA) fully abrogated binding in the relaxation-edited NMR assay. Binding was impaired but not abolished for the “central patch” mutant variant (L560W). We confirmed these results in the orthogonal waterLOGSY assay (Fig. 5D, Table 4).

**Table 4:** Ligand-observed NMR analysis to assess compound **9** binding to wild-type and mutant variants of TNKS2 ARC4.

TNKS2 ARC4 constructs	T2 relaxation-edited	waterLOGSY
compound alone	—	no NOE
wild-type	42.4% reduction	negative NOE
arginine cradle (WFE591/593/598AAA)	43.8% reduction	negative NOE
central patch (L560W)	22.4% reduction	positive NOE
aromatic glycine sandwich (YY536/569AA)	0%	positive NOE

### Fragment binding site mapping by protein-observed NMR

**Figure 6:**
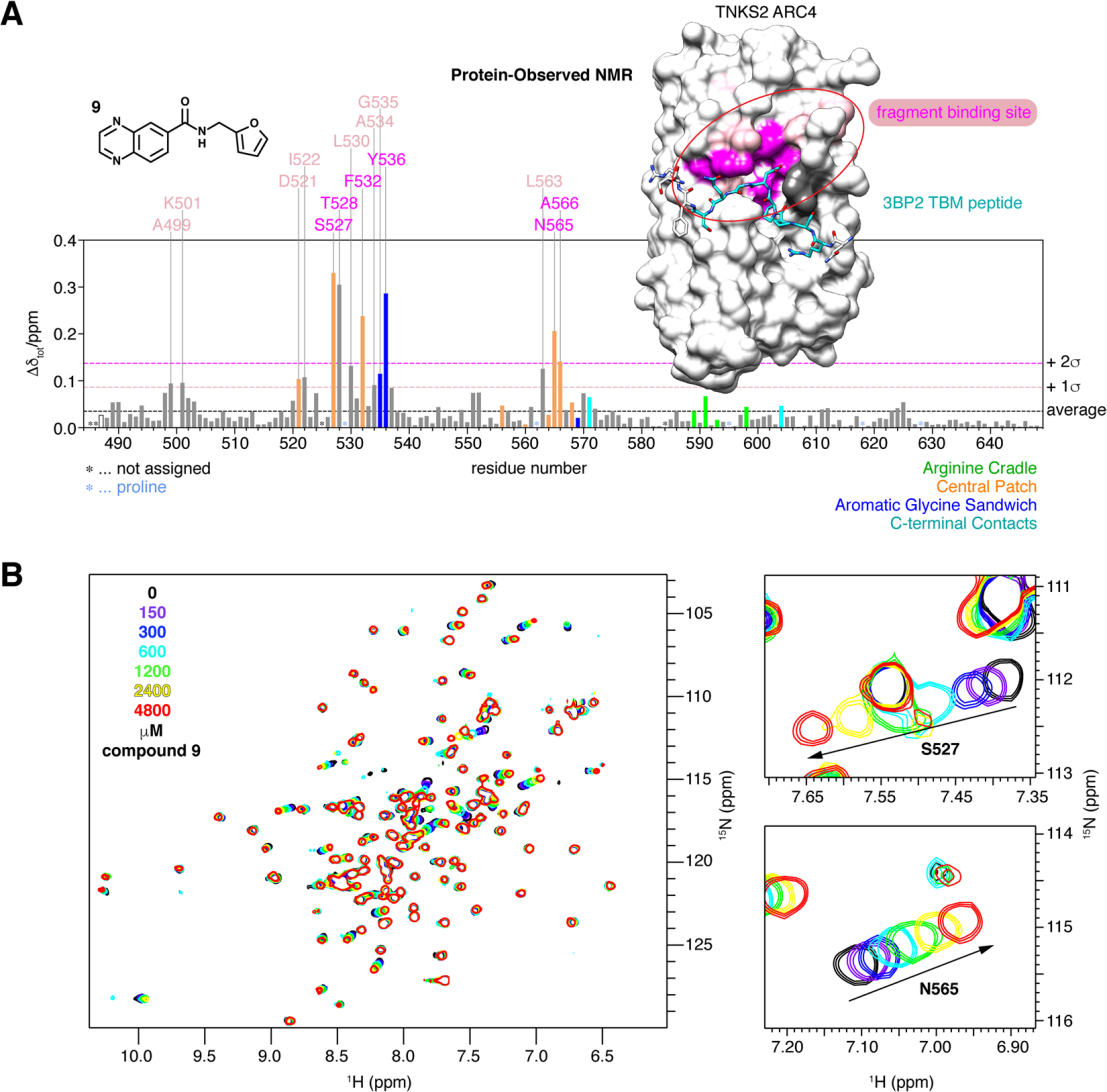
**(A)** Plot of CSPs in TNKS2 ARC4 (300 μM) induced by the addition of 16-fold excess (4800 μM) of compound **9** (see Fig. 4B,C). Bars corresponding to residues known to bind the TBM^4^ are colour-coded as in Figs. 1B and 5A,B. CSPs are mapped onto the surface representation of TNKS2 ARC4 bound to the TBM peptide of 3BP2 (shown in stick representation, 3TWR^4^): the strongest perturbations (>2σ of average) are shown in magenta, weaker ones (>1σ and ≤2σ) in pink. The overlap of the TBM binding pocket and compound **9**-induced CSPs is clearly apparent. Unassigned residues are shown in dark grey. Prolines are shown in light blue (none on the peptide-binding face of the ARC). **(B)** Whole ^1^H-^15^N HSQC NMR spectra of TNKS2 ARC4 and selected areas, showing the CSPs upon compound **9** titration.

To directly identify the compound **9** binding site on the ARC, we analysed the titrations of compound **9** with ^15^N-labelled TNKS2 ARC4 (see Fig. 4B,C). The higher solubility of fragment **9**, compared to that of compounds **3** and **5**, meant that much larger CSPs could be achieved (Supplementary Fig. 4C). At an ARC4:compound ratio of 1:16, close to signal saturation (see Fig. 4C, [compound **9**] = 4800 μM), we observed substantial CSPs for the following main-chain resonances: with CSPs above 2 σ from the mean CSP for S527, T528, F532, Y536, N565 and A566, and with CSPs within 1–2σ for A499, K501, D521, I522, L530, A534, G535 and L563 (Fig. 6). All CSPs occurred in the fast-exchange regime, and they included those observed for compounds **3** and **5** (Fig. 6, Supplementary Fig. 4C). Mapping the CSPs onto the crystal structure of TNKS2 ARC4 bound to the TBM peptide from 3BP2^4^ revealed the substantial overlap of the fragment and TBM peptide binding sites in the “aromatic glycine sandwich”, the “central patch” and residues in the close vicinity to these areas, in agreement with the mutagenesis studies (Fig. 6A). In conclusion, compound **9** occupies the most ligandable pocket on the ARC and a major binding hotspot of TBM peptides.

### *In-silico* fragment docking

**Figure 7:**
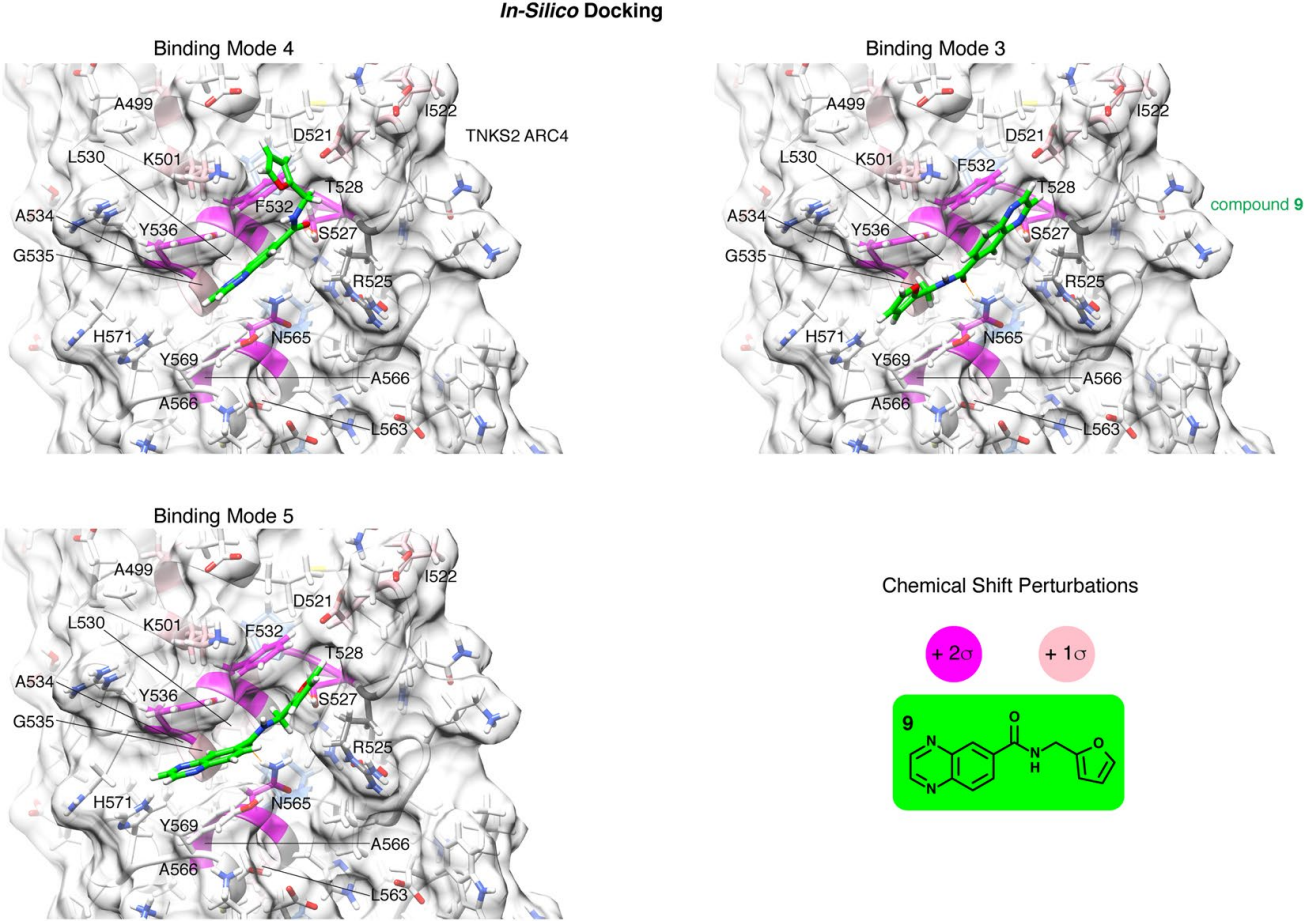
The three energetically most favourable binding modes of compound **9**, obtained by *in-silico* docking to TNKS2 ARC4 and FMO analysis. Each of the three binding modes encompassed several similar poses (see Table 5 and Supplementary Fig. 7A); the poses with the lowest TIE were selected as representatives for each binding mode. Selected key contact residues are labelled. Colouring is as in Fig. 6A. Residues coloured in magenta or pink represent those with strong (>2σ) and moderate CSPs (>1σ and ≤2σ), respectively, of the average CSP observed by protein-observed NMR with TNKS2 ARC4 (see Fig. 6). See Supplementary Fig. 7B for compound **9** binding modes superimposed with the TBM peptide from 3BP2, and Supplementary Fig. 7C for a 2D ligand-protein interaction diagram describing binding mode 4.

We next performed *in-silico* docking of fragment **9** to TNKS2 ARC4, aiming to satisfy the CSPs observed in our protein-observed NMR studies. We constrained the GOLD docking software to focus on a region within a distance of 14 Å from the PDGQS sequence of the 3BP2 TBM peptide (positions 4 to 8), as informed by protein-observed NMR. GOLD returned 18 binding poses, which upon visual inspection we clustered into eight distinct binding modes of similar binding poses (Table 5, Supplementary Fig. 7A). To identify the most likely, i.e. energetically most favourable, binding modes, we ranked the 18 poses by performing advanced *ab-initio* calculations using the fragment molecular orbital (FMO) method. This method quantitatively estimates the individual contributions and the chemical nature of each residue towards ligand binding. Therefore, the energy of binding can be evaluated for each binding hypothesis at a quantum-mechanical level (see Materials and Methods for details and references). We limit our discussion below to the top three binding modes, namely 4, 3 and 5, which the FMO method estimated to be energetically most stable (Table 5, Fig. 7, Supplementary Fig. 7). All three binding modes satisfy the large CSPs (>2σ) observed by compound **9** titration in protein-observed NMR, either by hydrogen bonds or van-der-Waals contacts, with the exception of T528, which is not directly contacted by the compound in any of the binding hypotheses.

**Table 5:** FMO total interaction energy (TIE) between compound **9** and TNKS2 ARC4, for 18 binding poses proposed by GOLD.

rank	binding pose	TIE
**1**	**BM4_C**	**−75.552**
**2**	**BM3_C**	**−70.805**
3	BM3_A	−67.648
4	BM4_B	−67.226
5	BM3_B	−67.056
6	BM4_D	−65.18
**7**	**BM5_A**	**−61.946**
8	BM5_B	−59.479
9	BM2_B	−58.031
10	BM4_A	−57.303
11	BM8	−55.717
12	BM6_A	−54.681
13	BM6_B	−52.393
14	BM2_A	−51.127
15	BM1_A	−47.69
16	BM1_B	−42.869
17	BM1_C	−38.941
18	BM7	−35.303

Binding poses cluster into eight binding modes. The three top-ranking binding poses for the three highest-scoring binding modes (BM4, 3, 5, shown in Fig. 7 and Supplementary Fig. 7) are indicated in bold.

In the top-scoring pose of binding mode 4, the pyrazine ring of the quinoxaline sits between the aromatic side chains of the “glycine sandwich” (Y536, Y569), whilst the rest of the compound occupies the adjacent “central patch” (Fig. 7). The breakdown of the total interaction energy (TIE) between compound **9** and TNKS2 ARC4 shows that compound **9** in binding mode 4 can strongly interact with Y536, Y569, G535, F532, S527 and R525; all these residues contribute to the TIE with a term lower than −5 kcal/mol. With the exception of Y569, which did not experience any CSPs, and R525, which was not assigned, the aforementioned residues showed substantial CSPs (moderate CSPs between 1 and 2σ for G535 and strong CSPs of >2σ for the others). A hydrogen bond between the compound **9** amide carbonyl and the S527 side chain mimics an equivalent contact involving the Asp residue at position 5 of the 3BP2 TBM model peptide (see Supplementary Fig. 7B). Binding mode 4 further satisfies moderate CSPs by interactions between the furan group with K501 and the furan and adjacent methylene with D521.

In binding mode 5, compound **9** is more extended and translated such that the quinoxaline group more extensively sits between the aromatic residues of the “glycine sandwich”. Hydrogen bonds are established between the amide carbonyl and N565 and between the furan oxygen and S527. Both these hydrogen bonds mimic those observed with the TBM peptide (see Supplementary Fig. 7B), and both N565 and S527 were characterised by large CSPs. FMO calculations estimate very favorable interactions (<−5 kcal/mol) of compound **9** with residues Y536, Y569, G535, N565, S527 and R525.

In binding mode 3, the fragment is flipped relative to its orientation in binding modes 4 and 5. Instead of the quinoxaline, the furan and adjacent methylene and amine are sandwiched by the aromatic residues of the “glycine sandwich”. Strikingly, binding mode 3 also features hydrogen bonds with N565 and S527. The hydrogen bond of the compound **9** amide carbonyl with the N565 side chain is preserved and serves as the “pivot point” of the flip relative to binding mode 5. The quinoxaline occupies the “central patch”, more specifically the space accommodating the Asp residue at position 5 of the 3BP2 model peptide and a water molecule^4^, hydrogen-bonding with the S527 side chain. FMO calculations indicate that highly favourable contacts are established with residues Y536, Y569, G535, N565, S527, R525 and D521.

In all three binding modes, the sensitivity of ARC binding to amide nitrogen methylation (see compound **14**, Table 1) may be explained by steric hindrance through the methyl group, which may induce a conformational change in the compound that is incompatible with ARC binding.

In conclusion, FMO calculations are in agreement with NMR observations, proposing binding modes 4, 3 and 5 as the most probable.

## Conclusions and Discussion

Here we identify a quinoxaline-based set of fragments that bind to the substrate/protein-binding ARCs of tankyrase at the same site as TBM peptides. We show that the fragments bind in the “aromatic glycine sandwich” and “central patch” regions, major known hotspots of TBM binding, and propose several possible binding modes. These fragments, even at their current affinities in the millimolar range, provide a potential starting point for the development of tool compounds to investigate the scaffolding roles of tankyrase, with the aim to validate whether tankyrase substrate binding antagonists are a viable approach to inhibiting tankyrase function.

Synthesising a set of TBM peptide variants as part of an initial peptidomimetic approach, we found that the essential, invariant arginine residue at TBM position 1 is challenging to replace with other groups that are less likely to impair cell permeability. Given that neither of the arginine substituents analysed here sufficiently preserved TBM binding, we took a fragment screening approach. Fragment screening circumvents potential challenges associated with the time-consuming, iterative optimisation of a peptide into a peptidomimetic and enables a diverse chemical space to be screened in an unbiased manner. Our *in-silico* analyses that preceded fragment screening point to the “central patch” region as the top-ranking, potentially ligandable pocket of the ARC. Indeed, the identified fragment binding site overlaps substantially with the “central patch” and the adjacent “aromatic glycine sandwich”, an anchor point for another invariant TBM residue, a glycine residue at TBM position 6. In protein-observed NMR studies, both the “central patch” and “aromatic glycine sandwich” coincide with TBM peptide-induced CSPs in the slow-exchange kinetic regime. This further confirms their critical role in TBM binding and illustrates that the fragments indeed target key determinants of the ARC:TBM interaction.

Effective substrate binding antagonists will likely need to target all TBM-binding ARCs in both tankyrase paralogues. Given the high degree of conservation between TNKS and TNKS2 ARCs, and the nearly identical TBM peptide binding infrastructure between different ARCs^4,48^ (Supplementary Fig. 6), this appears feasible. We indeed observe multi-ARC-binding activity of compound **9**. The non-detectable binding of this fragment to TNKS2 ARC1 may be a consequence of its overall low affinity for tankyrase. Multi-ARC binding will need to be monitored as more potent compounds are developed.

Future studies will focus on the structure-based design of TBM competitors with increased affinity. Fully developed tankyrase substrate binding antagonists will enable the complex mechanisms of tankyrase to be probed in a wide range of its biological functions. In the longer term, substrate binding antagonists may be of potential therapeutic value as they offer an opportunity to block both catalytic and non-catalytic functions and may display pharmacodynamics that substantially differ from compounds targeting the tankyrase PARP catalytic domain.

## Materials and Methods

### Protein expression and purification

Tankyrase ARC constructs were produced as previously described (see Pollock *et al*., 2017 for construct details)^49^. Uniformly ^15^N-labelled protein was produced in *E. coli* grown in M9 minimal media containing ^15^N ammonium chloride (CK Isotopes). One litre of M9 minimal media was prepared by combining M9 medium (10X stock, 100 mL), trace elements solution (100X, 10 mL), glucose (20% w/v, 20 mL), magnesium sulfate (1 M, 1 mL), calcium chloride (1 M, 0.3 mL), biotin (1 mg/mL, 1 mL), thiamin (1 mg/mL, 1 mL) and making up to 1 L with water. M9 medium (10X) contained disodium hydrogen phosphate (60 g/L), potassium dihydrogen phosphate (30 g/L), sodium chloride (5 g/L), and ^15^N ammonium chloride (25 g/L).

BL21-CodonPlus (DE3)-RIL *E. coli* cells were transformed with a pETM30-2 plasmid containing the gene for a His_6_-GST tagged human tankyrase ARC construct^4^. A single colony was selected and amplified in LB media (5 mL, Laboratory Support Services, ICR) for 6 h. This culture (1 mL) was then used to inoculate minimal media (200 mL) containing kanamycin (50 μg/mL) and chloramphenicol (34 μg/mL), and grown at 37 °C overnight. This starter culture (25 mL) was then used to inoculate each litre of minimal media, containing antibiotics as before. Cultures were grown at 37 °C with shaking (180 rpm) to an optical density of 0.6, measured at 600 nm. The temperature was reduced to 18 °C, and protein expression was induced by the addition of isopropyl-β-D-1-thiogalactopyranoside (IPTG) (0.5 mM). The cultures were incubated at 18 °C overnight. Cells were harvested by centrifugation (4000 × g, 30 min). The pellet was stored at −80 °C until purification following the previously described method^49^.

Doubly ^15^N/^13^C-labelled protein for the backbone and partial side-chain assignment of TNKS2 ARC4 was prepared as the ^15^N-labelled protein, except that ^13^C-D-glucose (Cambridge Isotope Laboratories; at 6 g/L of M9 media) was used as well. Method details have been reported elsewhere^61^.

### Fragment screening using a thermal shift assay

For the screen, a C1000 thermal cycler (Bio-Rad) was used to record melt curves. SYPRO Orange was purchased as a 5000 × stock in DMSO from Sigma Aldrich. The ICR fragment library was available as 100 mM stocks in DMSO, and dispensed (25 nL) using an ECHO acoustic liquid handling system into white 384-well PCR plates (Framestar, 4titude). Wells were backfilled with DMSO (225 nL). Buffer (2.75 μL, 25 mM HEPES-NaOH pH 7.5, 100 mM NaCl, 2 mM TCEP) was added, followed by TNKS2 ARC5 (1 μL, 100 μM stock) and then SYPRO orange dye (1 μL, 25×). The plate was centrifuged after the addition of each reagent (1 min, 1000 × g). Final assay concentrations were as follows: TNKS2 ARC5 (20 μM); fragment (500 μM); SYPRO Orange dye (5×); DMSO (5% v/v) in a total volume of 5 μL. Peptide control wells (3BP2 TBM 16-mer, 200 μM, sequence LPHLQ**RSPPDGQS**FRSW with C-terminal tryptophan added for photometric concentration measurements, the N-terminus acetylated and the C-terminus amide-capped) were plated in triplicate, and there were 12 blank wells per plate, with DMSO only (250 nL, 5% v/v). Melt curves were recorded from 20–95 °C, with the temperature ramped by 0.5 °C every 15 s. Data were analysed using Vortex (Dotmatics) software and melting temperatures calculated from both the inflection point and the maximum peak of 1^st^-derivative data. Data points were excluded if the melt curve was poor, i.e., if there was no fluorescence signal above baseline, high fluorescence intensity throughout, or if the melt curve was shallow (<1000 rfu difference between baseline and peak maximum). The unbound melting temperature was determined from the mean of 12 reference melting curves, with 5% DMSO only. The change in melting temperature (ΔT_m_) was calculated by subtracting the mean T_m, 0_ from T_m, compound_. Compounds were tested in duplicate, and fragments were defined as hits if they gave a ΔT_m_ outwith 2 σ from the mean in one or both replicates.

For the experiment shown in Fig. 2A, ARC and 3BP2 TBM peptide concentrations were 20 and 200 μM, respectively, in 50 mM HEPES-NaOH pH 7.5, 100 mM NaCl, 2 mM TCEP and a total volume of 25 μL. SYPRO Orange was added at 5×. Data were recorded from 4–95 °C, with the temperature ramped by 0.5 °C every 15 s, using a CFX384 thermal cycler (Bio-Rad). Data were analysed by non-linear regression in GraphPad Prism using a Boltzmann sigmoid with linear baselines. ΔT_m_ values were determined using the inflection point method.

### NMR experiments

A Bruker 500 MHz instrument, fitted with a 1.7 mm TXI microprobe, was used for all ligand-observed and protein-observed NMR experiments, with samples in 1.7 mm SampleJET NMR tubes (Bruker).

### Fragment solubility assay

Fragments were dispensed into a 384-well plate (250 nL of 100 mM stock in DMSO, 500 μM final concentration) using an ECHO acoustic dispenser. DMSO (2.25 μL) was added, then NMR buffer (47.5 μL, 25 mM HEPES-NaOH pH 7.5, 100 mM NaCl, 1 mM TCEP, 10% D_2_O). The plate was centrifuged (1 min, 1000 × g) before the solutions were transferred to 1.7 mm NMR tubes using a Gilson liquid handling system. ^1^H NMR spectra were recorded with the DMSO and water signals dampened. 100 μM caffeine was used as an external standard to quantify the ligand signals.

### T2 relaxation-edited NMR assay

Fragments were dispensed in duplicate into a 384-well plate (250 nL, 100 mM stock in DMSO). Wells were backfilled with DMSO (2.25 μL). Tankyrase ARC protein (47.5 μL in 25 mM HEPES, pH 7.5, 100 mM NaCl, 2 mM TCEP, 10% D_2_O; 20 μM final protein concentration in the assay) was added to the ‘protein’ samples; buffer only (47.5 μL) was added to the ‘compound-only’ samples. Solutions were transferred to 1.7 mm NMR tubes using a Gilson liquid handling system. ^1^H NMR spectra were recorded, with the DMSO and water signals dampened. A relaxation spin filter was applied at 400 ms^65^. Data were processed using Bruker Topspin 3.14. Lines were broadened with LB = 3.0, and the baseline was corrected between 6.0–10.0 ppm. The average integral for all peaks between 6.0–10.0 ppm was calculated, and the difference between compound-only and compound-plus-protein samples was compared. A reduction in signal integrals of ≥15% was classified as a hit. For competitive experiments, 3BP2 16-mer peptide (100 μM) was added, and the spectra were recorded and processed as above. The variability in signal reduction in the relaxation-edited experiment was previously determined as approximately ± 10% (Liu *et al*., unpublished observations). Therefore, replicates were run to account for this variability and to ensure that compounds that resulted in a weak signal reduction that did not meet the arbitrary cut-off were not erroneously excluded.

### WaterLOGSY NMR assay

Fragments were dispensed in duplicate into a 384-well plate (250 nL, 100 mM stock in DMSO). Wells were backfilled with DMSO (2.25 μL). Tankyrase ARC protein (47.5 μL in 25 mM HEPES-NaOH pH7.5, 100 mM NaCl, 2 mM TCEP, 10% D_2_O; 20 μM final protein concentration in the assay) was added to the samples containing protein; buffer only (47.5 μL) was added to the compound-only samples. Solutions were transferred to 1.7 mm NMR tubes using a Gilson liquid handling system. ^1^H NMR spectra were recorded, with the DMSO signal dampened. The bulk water signal at 4.7 ppm was selectively inverted. Data were processed using Bruker Topspin 3.14^66^.

### Fragment screening using a T2 relaxation-edited ligand-observed NMR assay

Cocktails of four structurally distinct compounds were created using MNova Screen software to ensure there was no significant overlap of peaks in the region of interest (5.5–9.5 ppm). Fragments were screened at 1 mM each, with 4% v/v DMSO. Compounds were dispensed in duplicate using an ECHO acoustic dispenser (0.5 μL of each, 100 mM stock in DMSO). TNKS2 ARC4 (35 μM in 25 mM HEPES-NaOH pH 7.5, 100 mM NaCl, 2 mM TCEP, 10% D_2_O) was added to one cocktail, and buffer alone to the other replicate for a control sample of compounds alone. Mixtures were incubated for 20 min at room temperature, and then transferred into 1.7 mm NMR tubes. ^1^H relaxation-edited NMR spectra were collected with double solvent suppression applied to dampen the water and DMSO solvent signals. ^1^H spectra of each individual compound were used as reference spectra.

Data were processed using Bruker Topsin 3.14, then analysed using the MNova Screen software. Only peaks between 5.5 and 9.5 ppm were considered. Peaks with a height of <5% maximum peak height within the region of interest were considered noise, and the minimum matched peak level was set at >51%. The relative peak intensity change (I) was calculated by Eq. 1 for all peaks in the 5.5–9.5 ppm region, for each compound.

1 $${\rm{I}}=({{\rm{I}}}_{{\rm{blank}}}-{{\rm{I}}}_{{\rm{protein}}})/{{\rm{I}}}_{{\rm{blank}}}$$

The average percentage change was then calculated, and compounds designated a hit if the signal was reduced by ≥26% (2 σ). Hit fragments were split into two groups: those with a signal change >39% (3 σ, 35 compounds), and those with a signal change of 26–39% reduction (2–3 σ, 65 compounds). Compounds of the first hit group (>3 σ) were tested individually in a second relaxation-edited assay, and the second hit group was tested in a waterLOGSY experiment, reasoning that this may rescue any genuine binders with a relatively small signal in the relaxation-edited assay, which has an intrinsic variability of ±10%. Protein ^1^H spectra of TNKS2 ARC4 (200 μM) were measured at 24 h intervals to ensure protein stability and folding for the duration of the screening experiments.

### Mass spectrometry

#### Fragment quality control

An Agilent 1200 Series HPLC coupled to an Agilent 6520 Quadrupole time of flight (qToF) mass spectrometer, fitted with an ESI/APCI multimode ionisation source, was used. All solvents were modified with 0.1% formic acid. Fragments (2 mM in DMSO) were injected (2 μL) onto a Purospher STAR RP-18 end-capped column (3 μm, 30 × 4 mm, Merck KGaA). Chromatographic separation was carried out over a 4-min gradient elution (90:10 to 10:90 water:methanol) at 30 °C. UV-Vis spectra were measured at 254 nm on a 1200 Series diode array detector (Agilent). The eluent flow was split, with 10% infused into the mass spectrometer. Eluent and nebulising gas were introduced perpendicular to the capillary axis, and applying 2 kV to the charging electrode generated a charged aerosol. The aerosol was dried by infrared emitters (200 °C) and drying gas (8 L/min of N_2_ at 300 °C, 40 psi), producing ions by ESI. Aerosol and ions were transferred to the APCI zone where solvent and analyte were vaporised. A current of 4 μA was applied, producing a corona discharge between the corona needle and APCI counter electrode, which produced ions by APCI. The multimode source operated in simultaneous APCI/ESI mode. During simultaneous APCI/ESI, ions from both ionisation modes entered the capillary and were analysed simultaneously. The fragmentor voltage was set at 180 V and skimmer at 60 V. Mass spectrometry data were acquired in positive ionisation mode over a scan range of m/z 160–950 with reference mass correction at m/z 622.02896 (Hexakis(2,2-difluoroethoxy)phosphazene). Data was analysed using MassHunter Qualitative Analysis B.06.00 (Agilent). Compound purity was calculated using the highest value of %UV (at 254 nm) or %TIC (total ion count).

### K_d_ determination using chemical shift perturbation

#### TBM peptide and fragment titrations

^15^N-labelled TNKS2 ARC4 (488–649) (300 μM final concentration; 5 μL of 3 mM stock) in NMR buffer (45 μL, 25 mM HEPES-NaOH pH 7.5, 100 mM NaCl, 1 mM TCEP, 10% D_2_O) was used as the baseline sample. Peptide titration samples (Table 6) were prepared by diluting the 16-mer 3BP2 peptide with NMR buffer (45 μL), then adding TNKS2 ARC4 (300 μM final concentration; 5 μL of 3 mM stock). Separate samples were prepared for each concentration point. Fragment titration samples (Table 7) were prepared by diluting fragments in NMR buffer (25 mM HEPES-NaOH pH 7.5, 100 mM NaCl, 1 mM TCEP, 10% D_2_O) and backfilling with DMSO to keep a constant DMSO concentration of 5%. ^15^N-labelled TNKS2 ARC4 (300 μM final concentration; 5 μL of 3 mM stock) was added. Protein with 5% DMSO alone was used as the baseline. Separate samples were prepared for each concentration point. ^1^H-^15^N HSQC spectra were acquired over 3 h, with 64 scans and a spectrum width of 16.00 ppm for ^1^H and 29.00 ppm for ^15^N. The pH of the peptide and fragment stocks (at 3 mM) was confirmed to exclude the possibility that peak shifts were due to changes in pH during the titration.

**Table 6:** Peptide concentrations for titration in protein-observed NMR. The TNKS2 ARC4 concentration was 300 μM.

[peptide] (μM)	50	150	300	600	900	1500
ratio peptide:protein	1:6	1:2	1:1	2:1	3:1	5:1

**Table 7:** Fragment concentrations for titration in protein-observed NMR. The TNKS2 ARC4 concentration was 300 μM.

[fragment] (μM)	75	150	300	600	1200	2400	4800
ratio fragment:protein	1:4	1:2	1:1	2:1	4:1	8:1	16:1

#### Analysis of protein-observed NMR data

Data were processed in Bruker Topspin 3.14 and analysed using CcpNmr Analysis software v2.4.2^67^.

To enable identification of peptide and fragment binding sites, a full backbone and partial side-chain assignment of ^15^N-^13^C-labelled TNKS2 ARC4 (488–649) was performed. Overall, out of 165 amino acids (construct + 3 N-terminal residues introduced by the cloning method), 164 residues were assigned, and backbone amides were missing for only two non-proline residues. Assignment details and methods have been reported elsewhere^61^.

Peaks that shifted were picked manually in each spectrum. The chemical shifts for each peak were measured and exported into Microsoft Excel, where the change in chemical shift from baseline was calculated for hydrogen and nitrogen shifts. The average Euclidean distance shifted (d) was then calculated using Eq. 2, weighting the different nuclei:

2 $${\rm{d}}=\surd \{1/2[{{\rm{\delta }}}_{{\rm{H}}}^{2}+({\rm{\alpha }}.{{\rm{\delta }}}_{{\rm{N}}}^{2})]\},{\rm{where}}\,{\rm{\alpha }}=0.14$$

Values of d were plotted against ligand concentration in GraphPad Prism, and fitted with Eq. 3:

3 $$\frac{\Delta {\delta }_{{\rm{obs}}}={\Delta {\rm{\delta }}}_{{\rm{\max }}}\{({[{\rm{P}}]}_{{\rm{t}}}+{[{\rm{L}}]}_{{\rm{t}}}+{{\rm{K}}}_{{\rm{d}}})-{[{({[{\rm{P}}]}_{{\rm{t}}}+{[{\rm{L}}]}_{{\rm{t}}}+{{\rm{K}}}_{{\rm{d}}})}^{2}-4{[{\rm{P}}]}_{{\rm{t}}}{[{\rm{L}}]}_{{\rm{t}}}]}^{1/2}}{2{[{\rm{P}}]}_{{\rm{t}}}}$$

K_d_ values were calculated for each peak that shifted individually. The mean of all shifting peaks was then calculated to give an apparent K_d_ value^68^.

### K_d_ determination using isothermal titration calorimetry

An ITC200 instrument (MicroCal) was used, fitted with a twisted syringe needle, stirring at 750 rpm. All solutions were degassed using a ThermoVac before use. The reference cell was filled with buffer (200 μL, 25 mM HEPES-NaOH pH 7.5, 100 mM NaCl, 1 mM TCEP, 1% v/v DMSO). The cell was filled with TNKS2 ARC4 (488–649) (200 μL, 200 μM in identical buffer as above). 20 injections (1 × 0.5 μL, then 19 × 2 μL) of compound **9** (5 mM, 1% v/v DMSO in buffer) were performed, with 180 s between injections. Blank correction was performed by titrating compound into buffer alone (25 mM HEPES-NaOH pH 7.5, 100 mM NaCl, 1 mM TCEP, 1% v/v DMSO) using the same injection protocol as above. The first injections from each run were discarded from data analysis. Data were analysed using Origin software with a one-site binding model. Titrations were repeated n = 5. Global analysis was performed using SEDPHAT software^69^.

### *In-silico* prediction of fragment hotspots and pockets on TNKS2 ARC4

For the FTMap analysis, TNKS2 ARC4 chain D from the TNKS2 ARC4-3BP2 co-crystal structure (PDB 3TWR)^4^ was submitted to the FTMap web server (ftmap.bu.edu) and analysed under protein-protein interaction mode, as detailed under the published conditions^62,63^.

For pocket identification using the Roll algorithm, TNKS2 ARC4 chain D from the TNKS2 ARC4-3BP2 co-crystal structure was submitted to the Pocasa 1.1 web server (altair.sci.hokudai.ac.jp/g6/service/pocasa/) and analysed with the following parameters: probe radius, 2 Å; single point flag, 16; protein depth flag, 18; grid size, 1 Å; atom type, protein.

### *In-silico* fragment docking

*In-silico* fragment docking was performed using the commercial docking software GOLD (version 5.6) distributed by CCDC (https://www.ccdc.cam.ac.uk). The structure of human TNKS2 ARC4 in complex with the TBM peptide from 3BP2 (PDB 3TWR, chain D)^4^ was used as a protein template. The protein was prepared by adding hydrogens using the protonate-3D function within the software MOE, from CCG (https://www.chemcomp.com). A flexible ligand docking protocol was used, setting the GOLD autoscale parameter to 3, and using the PLP scoring function. We constrained GOLD to focus on a region within a distance of 14 Å from the PDGQS sequence of the 3BP2 TBM peptide (positions 4 to 8), as informed by protein-observed NMR, and performed 25 docking runs. We further refined the GOLD docking poses within MOE, using a rigid receptor approach, and adopting the Amber10:ETH force field along with the R-field solvation model (dielectric constants set to 2 and 80). Duplicate binding hypotheses after refinement were discarded. The remaining binding hypotheses were clustered, upon visual inspection, into eight different binding modes. We consider a “binding mode” to represent a cluster of few similar “binding poses”. Binding mode 1 (BM1) included 3 binding poses, BM2 2 poses, BM3 3 poses, BM4 4 poses, BM5 2 poses, BM6 2 poses, BM7 1 pose, and BM8 1 pose (see Supplementary Fig. 7A and Table 5). All 18 binding poses underwent more advanced *ab-initio* calculations for accurate estimation of binding energy, as described in the next section.

### *Ab-initio* fragment molecular orbital calculations (FMO)

To describe the compound **9**:TNKS2 ARC4 interaction more quantitatively, we performed advanced *ab-initio* calculations using the fragment molecular orbital (FMO) method. The FMO method is a general *ab-initio* method that can be applied to studying large molecular systems, in particular when a standard quantum-mechanical treatment is unfeasible due to the computational demand. The FMO method can reduce the calculation time by dividing a large biological system into small and more computationally tractable fragments; a protein residue, a ligand, or a water molecule are examples of fragments. A number of successful applications of the FMO method for studying large biological systems have been published in the last decade^70–74^. Here, the protein residues were treated as different fragments, as well as compound **9**, even if it is not possible to establish a univocal 1:1 correspondence between an FMO fragment and a protein residue. Fragmentation methods for FMO calculations have been reviewed in detail^72,75^. Upon fragmentation of the biological system, the FMO procedure performed (1) self-consistent field (SCF) calculations for all fragments and all fragment pairs in the system, (2) evaluated general properties, such as energy and gradient, and (3) computed a pair interaction energy term (PIE) for all the fragment pairs. The PIE between two fragments is a sum of five terms: electrostatic, exchange repulsion, charge transfer, dispersion and solvation. A detailed mathematical description of the method has been reported^76,77^. Finally, the total interaction energy (TIE) between compound **9** and TNKS2 ARC4 was calculated as the sum of all individual PIEs of the ligand. Given the *ab-initio* treatment, atom polarisability, charge transfer and quantum effects are considered, and the protein:ligand interaction energy is accurately estimated. Solvation effects were also considered by the use of the polarisable continuum model (PCM)^77^. Note that the TIE of compound **9** is not the difference between the free energy of the protein:ligand complex and the relative free energies of the isolated elements, as it does not include any estimation of the entropy associated with the binding event. The TIE of compound **9** is rather an estimation of the strength of the interaction between the protein and the ligand in its bound state. The binding pose with the lowest TIE, within a specific binding mode, was chosen as representative of the specific binding mode (BM4_C, BM3_C, BM5_A).

We used the FMO^78^ code version 5.1 as implemented in the general *ab-initio* quantum chemistry package GAMESS^79^, version 2018 R1, developed at the AMES laboratory, Iowa State University (https://www.msg.chem.iastate.edu/gamess/). Calculations were carried out using the second-order Moller-Plesset perturbation theory (MP2) and the 6–31 G* basis set, with the addition of diffuse functions to the COO^−^ group. The PCM was used to treat the solvation effect. Input files were prepared with an MOE SVL script kindly provided by CCG, and the automatic fragmentation method implemented in the script was used for fragmenting the protein. FMO calculations only considered residues within a radius of 4.5 Å from the ligand.

### Structural representations

All structural representations were generated using UCSF Chimera^80^ (developed by the Resource for Biocomputing, Visualization, and Informatics at the University of California, San Francisco, supported by NIH P41-GM103311), unless indicated otherwise.

## Supplementary information

Supplementary Information
